# Duration of therapy for locally advanced pancreatic cancer: Does it matter?

**DOI:** 10.1002/cam4.3081

**Published:** 2020-05-05

**Authors:** Richard Tuli, John David, Stephanie Lobaugh, Zhigang Zhang, Eileen M. O'Reilly

**Affiliations:** ^1^ Department of Radiation Oncology, David M. Rubenstein Center for Pancreatic Cancer Research Memorial Sloan Kettering Cancer Center New York NY USA; ^2^ Department of Epidemiology‐Biostatistics Memorial Sloan Kettering Cancer Center New York NY USA; ^3^ Gastrointestinal Oncology Service Department of Medicine, David M. Rubenstein Center for Pancreatic Cancer Research Memorial Sloan Kettering Cancer Center New York NY USA

**Keywords:** chemotherapy, duration of chemotherapy, National Cancer Database, pancreatic cancer, radiotherapy

## Abstract

**Introduction:**

Evidence‐based recommendations on duration of multiagent systemic therapy for LAPC are lacking. Herein, we assess the impact of duration of combination systemic therapy on survival of patients with LAPC.

**Methods:**

The National Cancer Database was interrogated to identify patients with untreated LAPC diagnosed from 2004 to 2014. Patients treated with ≥ 1 month of multiagent chemotherapy (MAC) and ≥ 6 months of follow‐up were included. Kaplan‐Meier survival curves were generated to examine OS of each MAC duration group. Univariable and multivariable Cox proportional hazards regression was used to examine the association between OS with demographic and clinical variables. Statistical computations were performed using SAS Software Version 9.4.

**Results:**

Of the 3410 patients, 1114 met inclusion criteria. Median age was 64 years. Median treatment duration was 3.2 months (range 1‐19.8). Median follow‐up was 23.5 months (range 3‐120). Median OS of all patients was 9.4 months (95% CI: 8.7‐10.1). Median OS of patients receiving ≥ 1‐4 months, >4‐6 months and > 6 months of MAC was 8.4 months (95% CI: 7.7‐9), 10.2 months (95% CI: 9‐11.8), and 12.8 months (95% CI 11.6‐16). Twelve‐month survival was 37% for patients receiving ≥ 1‐4 months, 43% for > 4‐6 months, and 56% for > 6 months. Female sex (*P* = .02), higher median household income (*P* = .03), and longer duration of MAC (*P* < .001) were independently associated with improved OS following multivariable analysis.

**Conclusion:**

This analysis in LAPC patients suggests that combination systemic therapy regimens of 6 months or more may optimize survival outcomes. Further investigation on the duration of systemic therapy question in LAPC is needed.

## Graphical abstract

We present a succinct contemporary National Cancer Database analysis on the clinical impact of duration of multiagent chemotherapy for previously untreated locally advanced pancreatic cancer patients. Our data suggest that combination systemic therapy regimens of 6 months or more may optimize survival outcomes.

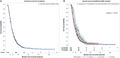

## 1 INTRODUCTION

Pancreatic adenocarcinoma (PDAC) remains the second leading cause of cancer deaths in the United States in spite of being the seventh most common malignancy.^1^ In 2019, an estimated 56 770 people were diagnosed with pancreatic cancer with 45 750 deaths.^1^ Poor patient outcomes are due to lack of screening, late presentation, high likelihood of occult metastatic disease, modest impact from the current best available therapies, and lack of targetable subtypes. Four driver mutations occur most frequently in PDAC (KRAS, p53, CDKN2A, SMAD4), yet none are considered therapeutically actionable. Gene expression and structural analyses have identified prognostic subgroups in PDAC, although as yet the clinical application is limited.^2^, ^3^, ^4^

Locally advanced pancreatic cancer (LAPC) patients have surgically unresectable primary tumors due to local arterial and/or venous vessel involvement. Neoadjuvant chemotherapy often followed by chemoradiotherapy or stereotactic body radiotherapy (SBRT) are standard of care treatments for LAPC yet result in poor clinical outcomes due to high rates of both metastatic and locoregional progression highlighting the imperative for better therapies.^1^ Recent advances in multiagent systemic therapy regimens for patients with metastatic PDAC have the potential to improve outcomes for patients with LAPC.^5^, ^6^ Phase 3 trials investigating multiagent chemotherapy regimens such as gemcitabine with nab‐paclitaxel and folinic acid, 5‐fluorouracil, inrinotecan, and oxaliplatin (FOLFIRINOX) in good performance‐status patients with metastatic PDAC have led to significant improvement in response rate, progression‐free, and overall survival compared with single agent chemotherapy.^5^, ^6^ Although these treatment combinations are being actively used in LAPC patients, there is limited data to guide the use of particular regimens as well as duration of such therapy.^7^

Duration of systemic treatment in metastatic PDAC is typically limited by disease progression or cumulative limiting toxicity related to fatigue, neuropathy, and myelosuppression. Furthermore, evidence‐based recommendations on duration of chemotherapy for LAPC are lacking and guided largely by treating physician's comfort and practice. The NCCN Guidelines recommend 4‐6 months of multiagent chemotherapy (FOLFIRINOX and gemcitabine/nab‐paclitaxel) largely based on older studies investigating single agent chemotherapy regimens.^7^, ^8^, ^9^ The potential detriment or benefit of treating with less than 4 months or more than 6 months of multiagent systemic therapy in LAPC are unknown. Herein, we assess the impact of duration of combination systemic therapy on survival of patients with LAPC.

## 2 METHODS

### 2.1 Database

The National Cancer Database (NCDB) was utilized to access de‐identified patient data with institutional review board review and oversight. The NCDB is a registry of data by the Commission on Cancer of the American College of Surgeons and the American Cancer Society. The NCDB includes data from more than 1500 facilities, encompassing approximately 70% of new cancers diagnosed in the United States. The included data points are outlined by the NCDB, which has established criteria to certify the quality of the submitted data and an application process. Following distribution of the data, the Commission on Cancer of the American College of Surgeons and the American Cancer Society have no oversight on the quality of the analyses.

### 2.2 Patient selection

The NCDB was interrogated to identify patients with treatment‐naïve clinical stage T4, node positive or negative, non‐metastatic PDAC diagnosed from 2004 to 2014 (AJCC 6th/7th Edition). Adenocarcinoma histology was defined as 8140‐41, 8145, 8154, 8210, 8230, 8255, 8260‐62, 8310, 8323, 8440, 8500, 8551, 8560, 8562, and 8570. International Classification of Diseases for Oncology‐3 codes for PC anatomic sites were included (C25.0‐C25.9). Multiagent chemotherapy was administered as the first course of therapy per NCDB definitions: Inclusion of patients treated with more than one chemotherapy agent at the same time and exclusion of patients treated with a single or unknown number of chemotherapeutic agents. Duration of chemotherapy was determined based on treatment start date until time of subsequent intervention. Patients treated with at least one month of multiagent chemotherapy (MAC) and at least six months of follow‐up post MAC initiation were included.

**Figure 1:**
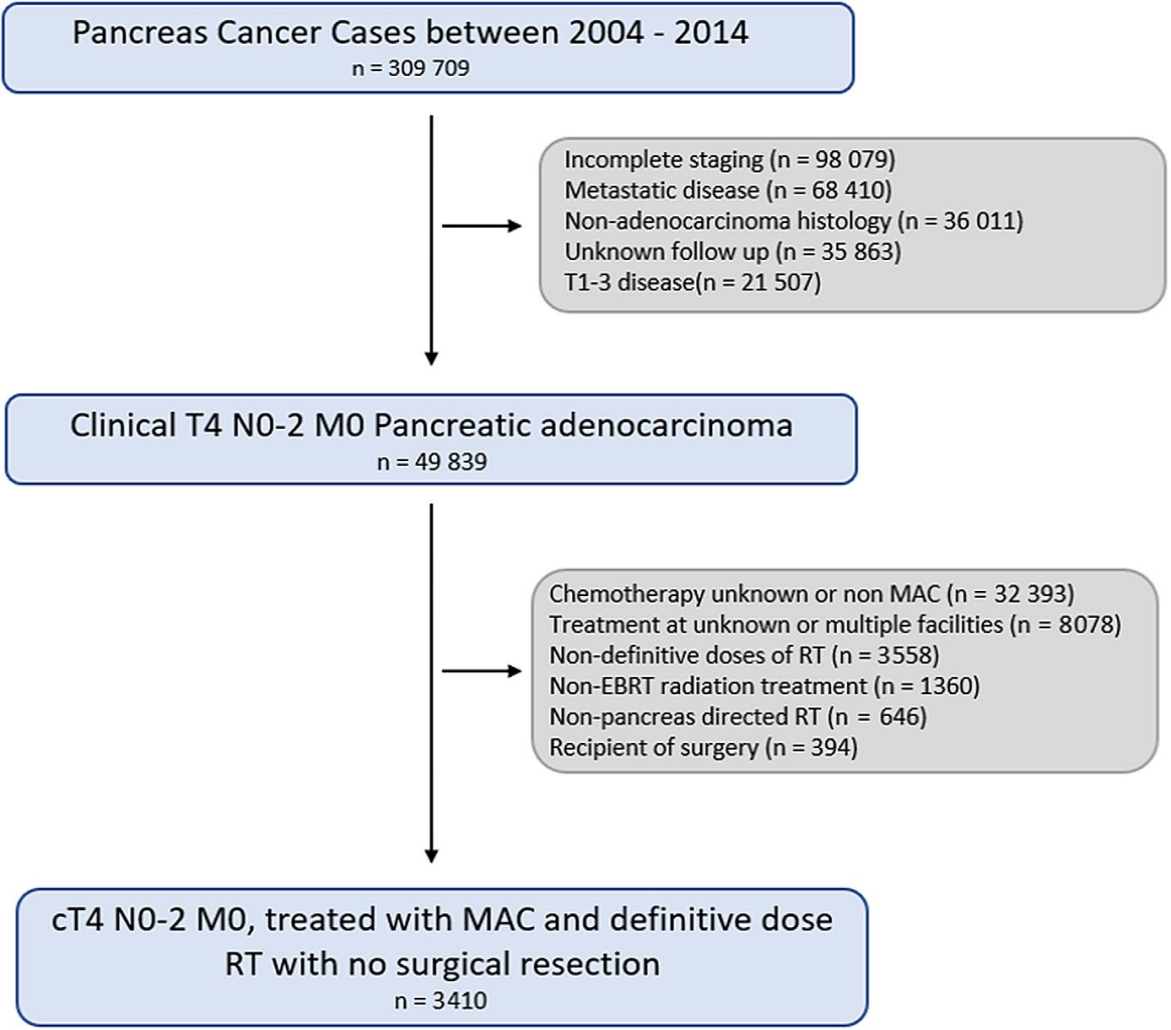
Consort Diagram of NCDB data extraction with excluded patients

Patients who received definitive doses of standard fractionated radiation (RT) (45‐65 Gy in 1.8‐2 Gy fractions) concurrent with chemotherapy, and stereotactic body RT (SBRT; 21 Gy in 3 fractions or 30‐50 Gy in 5 fractions), were included. Those who received radioactive implants, radioisotopes, combination external beam and radioisotopes, radiation type that was not otherwise specified, RT at multiple facilities, RT directed to nonprimary sites, nondefinitive dose of RT, unknown date from diagnosis to administration of RT, and those who had RT delivered prior to the administration of chemotherapy were excluded (see Figure 1) Patients were stratified according to duration of MAC: ≥1‐4 months, >4‐6 months, and > 6 months. Duration of MAC in days was converted to months using the following conversion factor: 30.417 days/month.

### 2.3 Statistical analysis

Survival time intervals were calculated from chemotherapy start date and landmarked at 6 months as each patient's MAC duration status was determined by this point. This was done to eliminate immortal time bias. A stratified Kaplan‐Meier survival curve was generated to examine the OS experiences of each MAC duration group. Univariable (UVA) Cox proportional hazards regression was also used to examine the association between OS and demographic and clinical variables. All variables were entered with MAC duration into a multivariable Cox proportional hazards model.

All statistical computations were performed, and all output was generated using SAS Software Version 9.4 (The SAS Institute, Cary, NC).

## 3 RESULTS

Of the 3410 patients included in the provided dataset, 2233 were excluded for receiving less than one month of MAC and 63 were excluded for less than six months of follow‐up. Median age was 64 years, 48% were female, 72% had Charleston/Deyo Comorbidity Index of 0.59% were N0, median duration of treatment cycles/months was 3.2 (range 1 to 19.8) and 49% received RT doses between 50 and 54 Gy.

Additional baseline characteristics, demographics, and univariate associations are presented in Table 1. There were significant differences in educational attainment (*P* = .01) and median household income (*P* = .005) between MAC duration groups. Patients who received MAC for longer duration of time (>4‐6 months and > 6 months) were more highly educated and had higher median income compared to patients who received a shorter duration (≥1‐4 months) of MAC. There were no other significant differences in patient characteristics when stratified by duration of MAC.

**Table 1:** Patient characteristics stratified by duration of MAC

	All patients	>1‐4 mo	>4‐6 mo	>6 mo	*P*‐value^a^
	N (%)	N (%)	N (%)	N (%)	
Sample size	1177	820 (69.7)	248 (21.1)	109 (9.3)	
Age at Dx					
Median (range)	64.00 (40.00‐89.00)	64.00 (40.00‐87.00)	64.00 (40.00‐89.00)	65.00 (41.00‐83.00)	>.99
N missing	(0)	(0)	(0)	(0)	
N =	1177	820	248	109	
Sex					
Male	617 (52.4)	440 (53.7)	126 (50.8)	51 (46.8)	.34
Female	560 (47.6)	380 (46.3)	122 (49.2)	58 (53.2)	
Education 2008‐2012^b^					
7%‐12.9%	407 (34.6)	275 (33.5)	95 (38.3)	37 (33.9)	.010
<7%	346 (29.4)	223 (27.2)	83 (33.5)	40 (36.7)	
13%‐20.9%	246 (20.9)	186 (22.7)	42 (16.9)	18 (16.5)	
21% or more	162 (13.8)	128 (15.6)	23 (9.3)	11 (10.1)	
Missing	16 (1.4)	8 (1)	5 (2)	3 (2.8)	
Median household income 2008‐2012^c^					
$63 000+	441 (37.5)	282 (34.4)	103 (41.5)	56 (51.4)	.005
$48 000‐$62 999	309 (26.3)	219 (26.7)	68 (27.4)	22 (20.2)	
$38 000‐$47 999	252 (21.4)	187 (22.8)	48 (19.4)	17 (15.6)	
<$38 000	158 (13.4)	123 (15)	24 (9.7)	11 (10.1)	
Missing	17 (1.4)	9 (1.1)	5 (2)	3 (2.8)	
CCI					
0	846 (71.9)	583 (71.1)	176 (71)	87 (79.8)	.27
1	270 (22.9)	196 (23.9)	58 (23.4)	16 (14.7)	
≥2	61 (5.2)	41 (5)	14 (5.6)	6 (5.5)	
T stage					
cT4	1177 (100)	820 (100)	248 (100)	109 (100)	
N stage					
cN0	693 (58.9)	480 (58.5)	147 (59.3)	66 (60.6)	.93
N+	484 (41.1)	340 (41.5)	101 (40.7)	43 (39.4)	
M stage					
cM0	1177 (100)	820 (100)	248 (100)	109 (100)	
Stage					
cStage III	1142 (97)	794 (96.8)	242 (97.6)	106 (97.2)	.33
cStage IV	13 (1.1)	10 (1.2)	2 (0.8)	1 (0.9)	
cStage IIA	8 (0.7)	6 (0.7)	2 (0.8)	0 (0)	
cStage IIB	3 (0.3)	1 (0.1)	0 (0)	2 (1.8)	
cStage II	1 (0.1)	1 (0.1)	0 (0)	0 (0)	
Missing	10 (0.8)	8 (1)	2 (0.8)	0 (0)	
Tumor grade					
Grade II,2,ii,I/III,1/3	108 (9.2)	71 (8.7)	21 (8.5)	16 (14.7)	.50
Grade III,3,iii,II/III,2/3	91 (7.7)	66 (8)	14 (5.6)	11 (10.1)	
Grade I,1,i	60 (5.1)	40 (4.9)	15 (6)	5 (4.6)	
Missing	918 (78)	643 (78.4)	198 (79.8)	77 (70.6)	
LVSI					
Absent/not identified	77 (6.5)	52 (6.3)	18 (7.3)	7 (6.4)	.41
Present/identified	24 (2)	13 (1.6)	9 (3.6)	2 (1.8)	
Missing	1076 (91.4)	755 (92.1)	221 (89.1)	100 (91.7)	
RT dose					
5000‐<5400	574 (48.8)	403 (49.1)	120 (48.4)	51 (46.8)	.90
≥5400	409 (34.7)	283 (34.5)	84 (33.9)	42 (38.5)	
4500‐<5000	194 (16.5)	134 (16.3)	44 (17.7)	16 (14.7)	

^a^ *P*‐values from Kruskal‐Wallis tests for continuous variables, from Chi‐square tests for education and income, and from Fisher's exact test for all other categorical variables.

^b^ Education 2008‐2012 ‐ This item provides a measure of the number of adults in the patient's zip code who did not graduate from high school, and is categorized as equally proportioned quartiles among all US zip codes.

^c^ Income 2008‐2012 ‐ Median household income for each patient's area of residence is estimated by matching the zip code of the patient recorded at the time of diagnosis against files derived from the 2012 American Community Survey data, spanning years 2008 2012 and adjusted for 2012 inflation. Household income is categorized as quartiles based on equally proportioned income ranges among all US zip codes.

**Figure 2:**
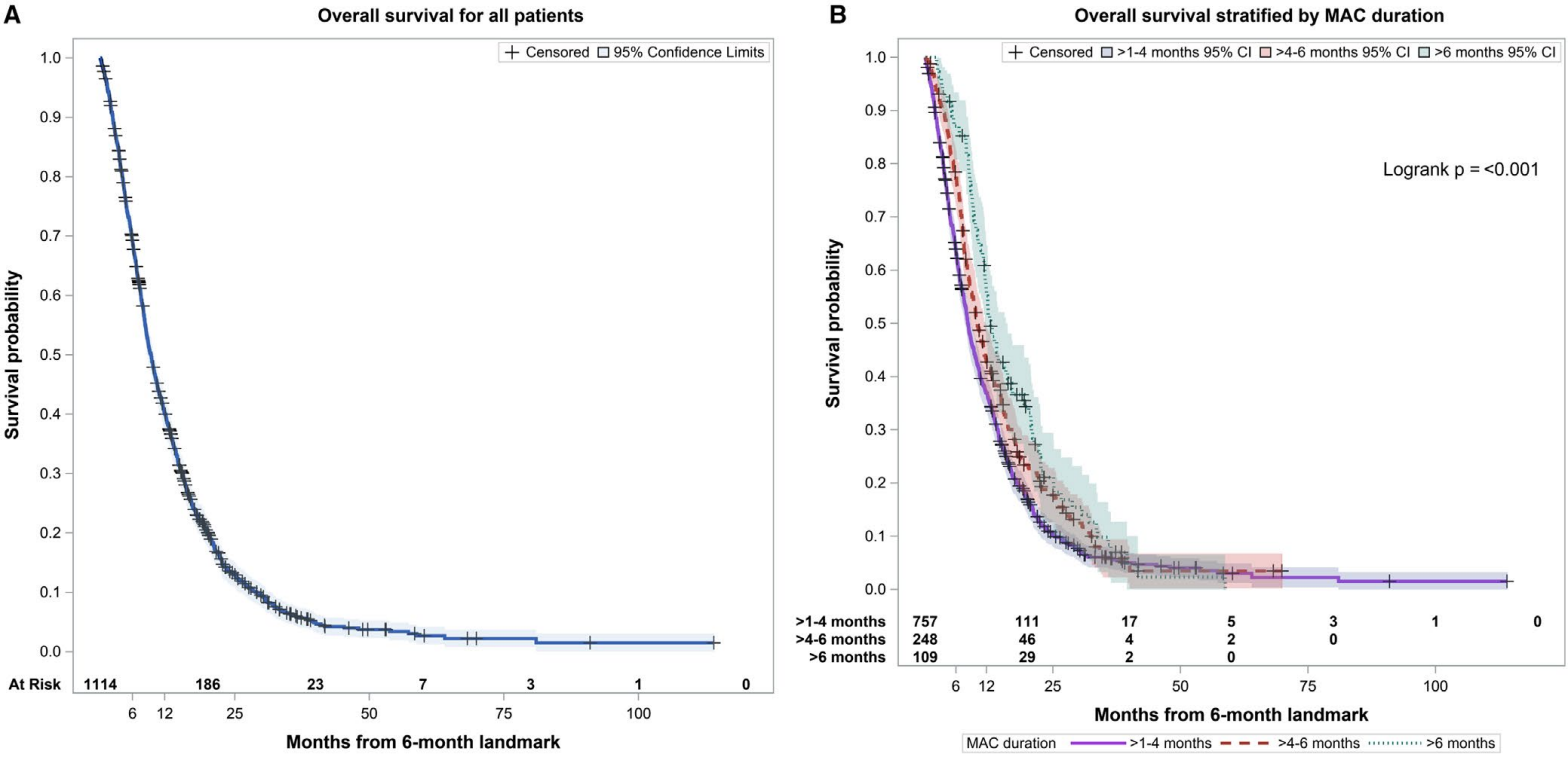
A, OS of all patients with median OS of 9.4 mo B, OS of patients stratified by duration of chemotherapy, 1‐4, 4‐6, and > 6 mo led to median OS of 8.4, 10.2, and 12.8 mo, respectively

Median follow‐up among survivors was 23.5 months with minimum and maximum follow up period of 3 months and 10 years, respectively. The number of patients receiving ≥ 1‐4 months, >4‐6 months, and > 6months of MAC was 757 (68%), 248 (22%), and 109 (10%), respectively. Median OS for the entire cohort was 9.4 months (95% CI: 8.7‐10.1; Figure 2(A)). Median OS of patients receiving ≥ 1‐4 months, >4‐6 months and > 6 months of 8.4 months (95% CI: 7.7‐9), 10.2 months (95% CI: 9‐11.8), and 12.8 months (95% CI 11.6‐16; Figure 2(B)). Twelve‐month survival was 37% (95% CI: 33%‐40%) for patients receiving ≥ 1‐4 months, 43% (95% CI: 36%‐49%) for patients receiving > 4‐6 months, and 56% (95% CI: 47%‐66%) for patients receiving > 6 months (Table 2).

**Table 2:** Overall survival estimates

Cohort	Estimate	
All patients	75th percentile in months (95% CI)	16.968 (15.980‐18.212)
	Median survival in months (95% CI)	9.407 (8.716‐10.062)
	25th percentile in months (95% CI)	4.840 (4.514‐5.462)
	6‐mo survival (95% CI)	0.690 (0.663‐0.718)
	12‐mo survival (95% CI)	0.401 (0.372‐0.430)
>1‐4 mo	75th percentile in months (95% CI)	15.817 (14.535‐16.900)
	Median survival in months (95% CI)	8.352 (7.729‐9.044)
	25th percentile in months (95% CI)	4.220 (3.722‐4.611)
	6‐month survival (95% CI)	0.637 (0.603‐0.671)
	12‐month survival (95% CI)	0.369 (0.334‐0.404)
>4‐6 mo	75th percentile in months (95% CI)	18.180 (16.181‐22.582)
	Median survival in months (95% CI)	10.161 (8.981‐11.807)
	25th percentile in months (95% CI)	6.422 (5.531‐7.207)
	6‐mo survival (95% CI)	0.773 (0.720‐0.825)
	12‐mo survival (95% CI)	0.427 (0.365‐0.490)
>6 mo	75th percentile in months (95% CI)	22.519 (20.252‐26.619)
	Median survival in months (95% CI)	12.788 (11.640‐16.110)
	25th percentile in months (95% CI)	8.920 (8.063‐10.292)
	6‐mo survival (95% CI)	0.871 (0.808‐0.934)
	12‐mo survival (95% CI)	0.561 (0.467‐0.656)

Abbreviation: CI, Confidence interval.

On univariable Cox regression analysis, female sex (*P* = .02) and longer duration of MAC (*P* < .001) were associated with improved OS (Table 3). In the fully adjusted multivariable model, female sex (*P* = .02), higher median household income (*P* = .03), and longer duration of MAC (*P* < .001) were independently associated with improved OS (Table 4). Administering > 4‐6 months or > 6 months of MAC led to HR of 0.8 (95% CI: 0.68‐0.94) and 0.66 (95% CI: 0.53‐0.82), respectively, compared to ≥ 1‐4 months of chemotherapy (Table 2).

**Table 3:** Baseline variable associations with overall survival UVA Cox proportional hazards regression

Variable	N (#Events)	HR (95% CI)	*P*‐value
Age at Dx	1114 (987)	1.00 (1.00‐1.01)	.36
Sex	1114 (987)		.02
Male	584 (524)	*Ref*.	
Female	530 (463)	0.86 (0.76‐0.98)	
Race	1102 (980)		.52
White	932 (834)	*Ref*.	
Black	138 (114)	0.90 (0.74‐1.10)	
Other	32 (32)	1.08 (0.76‐1.54)	
Median household income 2008‐2012	1098 (971)		.16
$63 000+	418 (366)	*Ref*.	
<$38 000	152 (137)	1.15 (0.94‐1.40)	
$38 000‐$47 999	240 (211)	1.11 (0.93‐1.31)	
$48 000‐$62 999	288 (257)	1.19 (1.02‐1.40)	
Education 2008‐2012	1099 (972)		.38
<7%	334 (298)	*Ref*.	
21% or more	152 (131)	0.89 (0.73‐1.10)	
13%‐20.9%	233 (205)	1.07 (0.90‐1.28)	
7%‐12.9%	380 (338)	1.04 (0.89‐1.22)	
MAC duration	1114 (987)		<.001
>1‐4 mo	757 (681)	*Ref*.	
>4‐6 mo	248 (213)	0.80 (0.68‐0.93)	
>6 mo	109 (93)	0.65 (0.52‐0.81)	
N stage	1114 (987)		.24
cN0	664 (577)	*Ref*.	
N+	450 (410)	1.08 (0.95‐1.22)	
CCI	1114 (987)		.78
0	803 (720)	*Ref*.	
1	256 (219)	1.00 (0.86‐1.16)	
≥2	55 (48)	1.11 (0.83‐1.49)	
RT dose	1114 (987)		>.99
4500‐<5000	171 (154)	*Ref*.	
5000‐<5400	555 (490)	1.00 (0.84‐1.20)	
≥5400	388 (343)	0.99 (0.82‐1.20)	

Abbreviations: CI, Confidence interval; HR, Hazard ratio.

**Table 4:** Baseline variable associations with overall survival MVA Cox proportional hazards regression

Variable	HR (95% CI)	*P*‐value
Age at Dx	1.00 (1.00, 1.01)	.49
Sex		.02
Male	*Ref*.	
Female	0.85 (0.75, 0.97)	
CCI		.54
0	*Ref*.	
1	0.99 (0.85, 1.16)	
≥2	1.18 (0.87, 1.60)	
N stage		.14
cN0	*Ref*.	
N+	1.10 (0.97, 1.26)	
MAC duration		<.001
>1‐4 mo	*Ref*.	
>4‐6 mo	0.80 (0.68, 0.94)	
>6 mo	0.66 (0.53, 0.82)	
RT dose		.95
4500‐<5000	*Ref*.	
5000‐<5400	1.02 (0.85, 1.23)	
≥5400	1.03 (0.85, 1.25)	
Race		.46
White	*Ref*.	
Black	0.88 (0.71, 1.09)	
Other	1.05 (0.73, 1.52)	
Education 2008‐2012		.09
<7%	*Ref*.	
21% or more	0.71 (0.54, 0.94)	
13%‐20.9%	0.90 (0.72, 1.12)	
7%‐12.9%	0.97 (0.81, 1.15)	
Median household income 2008‐2012		.03
$63 000+	*Ref*.	
<$38 000	1.45 (1.11, 1.88)	
$38 000‐$47 999	1.21 (0.98, 1.49)	
$48 000‐$62 999	1.22 (1.03, 1.46)	

Abbreviations: CI, Confidence interval; HR, Hazard ratio.

## 4 DISCUSSION

Duration of treatment with multiagent chemotherapy for LAPC is limited by disease biology and treatment related toxicity without clear knowledge regarding how therapy duration impacts overall survival. To the best of our knowledge, this is the first study demonstrating that a longer duration of combination systemic therapy is independently associated with OS in treatment naïve LAPC. The use of induction chemotherapy for LAPC as a means of sterilizing micrometastatic disease and controlling the primary tumor and regional lymph nodes has become a well‐established standard of care strategy in the absence of prospective data. Although locoregional progression in patients with LAPC can result in significant morbidity and mortality,^10^ the vast majority of patients progresses distantly and succumb to metastatic disease even during treatment with aggressive systemic therapy regimens.

Prior to the advent of contemporary multiagent regimens, disease progression typically occurred before any durable treatment response could be achieved with gemcitabine or 5‐FU alone, and limited data are therefore available on how much chemotherapy should be administered. In a seminal older study by,^11^ gemcitabine led to improved clinical benefit response and modest improvement in survival compared to 5‐FU in patients with LAPC or metastatic pancreatic cancer. Although treatment with either drug was planned to continue indefinitely until disease progression or intolerance, median PFS in this treatment naïve population with gemcitabine was only 9 weeks compared to 4 weeks with 5‐FU. Subsequent studies investigating gemcitabine combination strategies in LAPC and metastatic pancreatic cancer were also designed to continue treatment indefinitely until intolerance or progression.^12^ In this study, gemcitabine and cisplatin led to median PFS of 5 months vs 3 months with gemcitabine alone, yet median duration of treatment was only 4 vs 3 months, respectively in both arms. Treatment related grade 3 or 4 hematologic toxicities was approximately 15% in both arms. As disease control has improved with more aggressive multi agent chemotherapy regimens, treatment related toxicities have played a bigger role in limiting duration of treatment. In a phase 2 single arm study of FOLFIRINOX in advanced PDAC in which 76% of patients had metastatic disease, median PFS was a very promising 8 months with grade 3/4 neutropenia identified in 52% after a median of 8 cycles per patient.^13^ Likely a result of balancing encouraging efficacy with significant toxicity, six months of chemotherapy was recommended in the follow up phase 3 study of FOLFIRINOX versus gemcitabine in patients with metastatic PDAC. The median number of treatment cycles administered was 10 (5 months; range, 1 to 47) in the FOLFIRINOX group and 6 (6 months; range, 1 to 26) in the gemcitabine group (*P* < .001). Fewer patients in the FOLFIRINOX arm had disease progression prior to 6 months of treatment (55%) and median PFS was 6.4 months with grade 3/4 neutropenia occurring in a slightly lower (46%) proportion of patients.

Similar study designs incorporating six months of treatment have been extrapolated to the LAPC setting. The LAP07 study investigated 4 months of gemcitabine with or without erlotinib.^8^ In the absence of disease progression, participants were randomized to an additional 2 months of chemotherapy or 1.5 months of chemoradiotherapy. PFS ranged from 8 to 10 months, and interestingly, the median delay to treatment reintroduction was 6 months for the chemoradiotherapy group, which was significantly longer than the 4 months for the chemotherapy group (*P *= .02). This increased durability of tumor control seen in the locally advanced setting suggests investigation of duration of chemotherapy may be further warranted. In our study, increasing duration of multiagent chemotherapy to > 4‐6 months or >6 months led to significant increases in median OS of approximately 2 and 5 months compared to patients receiving ≥1‐4 months of MAC. As a point of reference, there was no improvement in OS following addition of erlotinib or chemoradiation to 6 months of gemcitabine alone in LAP07.^8^ A meta‐analysis combining patient‐level data from 11 studies with 315 LAPC patients treated with FOLFIRINOX indicated a pooled median OS of 24 months, which was significantly longer than previous reports investigating single agent regimens as well as those reported in our study investigating varying multiagent regimens. This study serves to highlight the benefit of using an aggressive multiagent regimen in good performance status LAPC patients this setting and further highlights the need for prospective randomized trials which are ongoing.^14^ The median number of administered cycles in the study by Suker et al as reported in nine of eleven studies ranged from 3‐11 cycles months and no correlation with median OS was identified (*P* = .95).^15^ Although the results differ from those identified in our study, actual duration of chemotherapy ranged from median of 1.5‐5.5 months and more extended regimens were not explored. Taken together, these data suggest that protracted duration of regimens such as FOLFIRINOX should be further explored in good performance status LAPC patients not experiencing dose limiting toxicities.

In a retrospective study of borderline resectable and LAPC patients treated with neoadjuvant gemcitabine/nab‐paclitaxel (3 weeks on, 1 week off/cycle) or FOLFIRINOX (every 2 weeks/cycle) followed by chemoradiation, Truty et al found that administering more than 6 cycles of chemotherapy (3‐6 months) led to significant improvements in recurrence free survival (27 months) and OS (60 months).^16^ Such exceptional clinical outcomes were very likely driven by the much higher proportion of borderline resectable patients (63%) in this study especially since only 37% of patients received > 8 cycles of chemotherapy and 91% of patients underwent resection with 94% margin negative rate. In stark contrast, patients in our study treated with > 6 months of MAC had a median OS of 13 months.

In spite of aggressive neoadjuvant strategies in LAPC incorporating both multiagent chemotherapy with or without chemoradiotherapy (total neoadjuvant therapy – TNT), survival rates have only incrementally improved largely due to distant failure but also failure to realize any demonstrable radiographic downstaging of the primary tumor. Recent meta analyses indicated resection rates ranging from 4% to 25%^8^, ^15^ in LAPC treated with induction chemotherapy and a large proportion of these patients still undergo margin positive resections as a result of inappropriate radiographic selection.^17^, ^18^ The promising findings identified in our study and others lend further support to treatment of resectable pancreatic cancer with neoadjuvant multiagent chemotherapy to sterilize occult metastatic disease present in a large proportion of patient and to allow selection of those patients who may realize the most significant benefits from locoregional therapy. This strategy is being explored in ongoing randomized clinical trials.^19^, ^20^

There are significant limitations to this study which are inherent to interrogation of the NCDB and retrospective studies. Duration of chemotherapy is likely influenced by a multitude of factors and biases not captured in the database such as type and combination of chemotherapy, frequency and severity of treatment‐related adverse events, availability, and administration of appropriate supportive care, and patient and/or physician decision‐preferences. To account for the different multiagent chemotherapy regimens available as first‐line options and associated variability in dosing and duration of individual cycles, we utilized months of treatment as the primary variable of investigation. Results may be further biased by the selection process of patients chosen to receive single versus multiagent chemotherapy and those who received longer versus shorter duration of treatment. To this end, the NCDB does not provide information on patient performance status and to our knowledge, there is no prognostic risk tool which has been validated for patients with pancreatic cancer. CCI was utilized as a surrogate for comorbidities but was not prognostic of survival. This study is also limited by inability to control for treatments received after TNT. All patients in our study received consolidative therapeutic doses of stereotactic body radiotherapy or standard fractionated chemoradiation after MAC. Inclusion of radiation was necessary to determine duration of MAC within the NCDB. Although it is possible that inclusion of radiation influenced clinical outcomes in this study, it is unlikely given the mixed data regarding clinical benefits of radiation in LAPC.^8^, ^9^, ^21^ Definitions for what constitutes locally advanced disease are not consistent amongst guidelines^7^, ^22^ and subject to significant interobserver variability.^22^, ^23^ In spite of this, clinical outcomes noted in this study are in line with historic controls investigated prospectively in LAPC.^8^, ^9^

### 4.1 Summary

An analysis of the NCDB in LAPC suggests that combination systemic therapy regimens of 6 months or more may optimize survival outcomes. Further investigation on the duration of systemic therapy question in LAPC is needed.

## DISCLOSURES

The authors have no conflicts of interest related to this work.

## AUTHOR CONTRIBUTION

All authors contributed equal in the conceptualization and design, collection and assembly of reference, interpretation, and writing. Stephanie Lobaugh was responsible for statistical analysis of the collected data.

## Data Availability

Data are available via National Cancer Database website at (https://www.facs.org/quality‐programs/cancer/ncdb).
